# An increase in intracellular p62/NBR1 and persistence of *Burkholderia mallei* and *B. pseudomallei* in infected mice linked to autophagy deficiency

**DOI:** 10.1002/iid3.239

**Published:** 2018-12-19

**Authors:** Kamal U. Saikh, Jennifer L. Dankmeyer, Xiankun Zeng, Robert G. Ulrich, Kei Amemiya

**Affiliations:** ^1^ Department of Immunology Army Medical Research Institute of Infectious Diseases Frederick Maryland USA; ^2^ Department of Bacteriology Army Medical Research Institute of Infectious Diseases Frederick Maryland USA; ^3^ Department of Pathology Army Medical Research Institute of Infectious Diseases Frederick Maryland USA

**Keywords:** autophagy, *Burkholderia mallei*, *Burkholderia pseudomallei*, p62, NBR1, intracellular survival

## Abstract

**Introduction:**

*Burkholderia mallei* (*B. mallei*) and *Burkholderia pseudomallei* (*B. pseudomallei*), causative agents of glanders and melioidosis, respectively, are invasive intracellular pathogens that actively multiply in phagocytic and non‐phagocytic cells. Activation of cell‐autonomous autophagy mechanism eliminate intracellular pathogens in which p62 a cytosolic cargo protein is selectively degraded, and an accumulation of this marker occurs if autophagy is deficient. Recurrent, relapsed and reinfection of *B. pseudomallei* in melioidosis patients in endemic area indicative of lack of complete of clearance and persistence of the pathogen. Reasoning that abundance in the levels of p62 may provide an indication of the intracellular infection, we sought to examine whether increase in intracellular p62 and bacterial burden with *Burkholderia* infection are linked to autophagy deficiency.

**Methods:**

In this study, we investigated cell culture and mouse models of disease to identify an association between autophagy biomarkers (p62/NBR1) accumulation and intracellular persistence of *B. mallei* and *B. pseudomallei*.

**Results:**

We demonstrate, that elevated levels of intracellular p62/NBR1 correlated with bacterial persistence, while pre‐treatment with a pharmacological inducer of autophagy, rapamycin, reduced both intracellular p62, and bacterial survival. Our results showed an elevated p62 levels (2‐5 fold) in spleen and liver cells of *Burkholderia*‐infected BALB/c mice, as well as in spleen cells of *Burkholderia*‐infected C57BL/6 mice, suggesting that an increase in p62/NBR1 was due to an autophagy deficiency. Similar to p62, cytosolic LC3‐I levels were also elevated, while the characteristic conversion to the autophagosome‐associated membrane bound form LC3‐II was low in spleens of the infected mice further supporting the conclusion that autophagy was deficient.

**Conclusion:**

Taken together, our results suggest that an increase in intracellular p62/NBR1 may be a potential host cell biomarker of *B. mallei* or *B. pseudomallei* infections, and identifying autophagy manipulation may potentially aid to therapeutic approach for complete clearance of the pathogen.

## 1 INTRODUCTION

Autophagy is an active cellular degradative process that removes or recycles bulk cytoplasmic constituents and intracellular pathogens through the endosomal and fusion system, resulting in the formation of autophagosomes in eukaryotic cells. Because this cellular process is induced by intracellular bacteria, the secretion of bacterial products, or phagosomal membrane damage, autophagy serves an important role as an innate intracellular defense mechanism against certain invasive pathogens.^1^ While autophagy results in the clearance of some bacterial pathogens from cytosol, others are able to manipulate this innate immune response for their own benefit, and appear to effectively replicate within autophagosome‐like vesicles. *Burkholderia mallei* (*B. mallei*) and *Burkholderia pseudomallei* (*B. pseudomallei*), causative agents of glanders and melioidosis, respectively, are facultative intracellular pathogens that are able to actively invade and multiply in phagocytic and non‐phagocytic cell lines of humans and animals.^2^, ^3^, ^4^, ^5^, ^6^ *B. thailandensis* is a non‐pathogenic soil saphrophyte, but closely related species to *B. pseudomallei* which is also present in the environment including geographic areas that are positive for *B. pseudomallei*.^7^ Both *B. mallei*, which is primarily a veterinary disease, and *B. pseudomallei*, which can infect human and most mammals, are listed as select agents by the U. S. Centers for Disease control and Prevention (CDC), due to the high risk of aerosol infection and because there is potential for misuse of both organisms as agents of biological warfare or terrorism. To date no effective human vaccines are available and current antibiotic therapy may be ineffective against chronic melioidosis.

Following cellular uptake, *B. pseudomallei*, is initially observed in vacuoles and later in the cytoplasm.^5^, ^6^ Dormant or inapparent infections occur and may recrudesce in severe fulminant form months and even years after exposure.^4^ Recurrent or relapsed *B. pseudomallei* infection of melioidosis patients in endemic areas may suggest a failure to clear an infection due to deficiency in innate immune effector mechanisms such as autophagy.^8^ Additionally, diagnosis of exposure to these pathogens appears to be challenging due to varied clinical presentations. It remains unclear if components of the autophagic machinery in infection by *Burkholderia spp*., may reveal useful information for disease diagnosis and treatment.

Autophagy is an evolutionarily conserved degradative and recycling pathway that plays a role in the cellular response to starvation,^9^ host‐defense through degradation of invading bacteria, and promoting cell survival bykilling intracellular bacteria.^10^, ^11^ The process begins with the sequestration of invading pathogens into double‐membrane vesicles (autophagosome), which then fuse with lysosomes, forming single‐membraned autophagolysomes, ultimately leading to the degradation of the autophagosomal contents. Cytosolic microbes are engulfed into double membrane autophagosomes or LC3‐associated phagosomes (LAP) that are guided by several pathogen recognition receptors (PRRs), including sequestosome 1/p62‐like receptors (SLRs). Ubiquitylated targets are linked to autophagosomal degradation pathways^12^, ^13^, ^14^ by p62 and other SLR that contain multiple cargo recognition domains that bind ubiquitin and have dual functionalities as both a scaffold protein and in trafficking for protein degradation, including invading pathogens. Most cellular p62 is found in the cytoplasm, but also localizes to the nucleus, as well as to autophagosomes and lysosomes. In response to various stressors, p62 translocates to protein aggregates, damaged mitochondria, infected bacteria, and similar autophagy substrates. Neighbor of BRCA1 gene 1 (NBR1) is also an autophagy receptor that has LC3 and ubiquitin (Ub)—binding domains. Both p62 and NBR1 act as cargo receptors for selective autophagosomal degradation of ubiquitylated targets, and are continuously cleared from the cytoplasm along with the associated cargo. Observations with the macrophage RAW264.7 cells infected with *B. pseudomallei* indicated that only a subset of bacteria co‐localized with the autophagosomal marker protein LC3.^15^ Further, electron microscopic images of ex vivo culture‐infected cells revealed that intracellular *B. pseudomallei* are either free in the cytosol or sequestered in single‐membrane phagosomes, but rarely contained in double‐membrane autophagosomes,^16^ suggesting that most bacteria escape to the cytosol to evade capture by canonical autophagy. In this study, we investigated cell culture and mouse models of disease to identify an association between autophagy biomarkers and intracellular persistence of *B. mallei* and *B. pseudomallei*.

## 2 MATERIALS AND METHODS

### 2.1 Ethics statement

Research was conducted under an IACUC approved protocol in compliance with the Animal Welfare Act, PHS Policy, and other federal statutes and regulations relating to animals and experiments involving animals. The facility where this research was conducted is accredited by the Association for Assessment and Accreditation of Laboratory Animal Care, International and adheres to principles stated in the 8th Edition of the Guide for the Care and Use of Laboratory Animals, National Research Council, 2011.

### 2.2 Reagents

Rapamycin was purchased from LC Laboratories (Woburn, MA). HeLa cells were purchased from ATCC (Manassas, VA). The p62 and NBR1 assay kit was purchased from Enzo Life Sciences (Framingale, NY). The Rabbit polyclonal p62 and NBR1antibody was purchased from Cell Signaling Technology (Danvers, MA) and ThermoFisher scientific (Grand Island, NY). For in situ hybridization RNAscope RED 2.5 kit (Cat# 322360) was purchased by Advanced Cell Diagnostics (ACD) and 20 ZZ probes against mouse SQSTM1/p62 (Cat# 444221) were synthesized and purchased from ACD.

### 2.3 Bacterial strains

The *B. mallei FMH* 23344 strain used in this study was obtained from Dr. D. Waag; *B. thailandensis and B. pseudomallei* Bp K96243, Bp 406e, Bp 1106A, and Bp HBPUB10134a were obtained from the Critical Reagents Program (US Army Research Institute of Infectious Diseases, Frederick, MD). The clinical isolates of *B. pseudomallei* were initially described in refs.,^17^, ^18^ and the original isolate of *B. mallei* FMH was described previously in ref.^18^

### 2.4 Mouse infections

A 4‐6 weeks old BALB/c and C57BL/6 used for this study were purchased from the National Cancer Institute, Frederick, MD. The aerosol infected mice were part of study to determine the median lethal dose 50% (LD_50_) of different human clinical isolates of *B. pseudomallei* and *B. mallei* FMH, and the selected bacterial strains were grown in 4% glycerol in 1% typtone broth (GTB) (Difco, Becton Dickinson, Sparks, MD) to late log at 37°C, as described previously.^19^ Mice were infected with escalating doses by whole‐body aerosol as described previously with a three‐jet Collison nebulizer, and surviving mice after 21 days post‐infection were evaluated.^20^ Criteria for early endpoint euthanasia were used during the infection studies. Mice were anesthesized and exsanguinated before removing the spleens for studies described here and below. The isolated mouse spleen cells were chilled on ice for 5 min before being pelleted into fresh 1.5 mL centrifuge tubes. Cells were lysed in 50 μL of lysis buffer (Active Motif) in the presence of DTT, protease inhibitors and phosphatase inhibitors and incubated on ice for 30–60 min. The membrane fraction was collected by centrifuging the lysates at 14 000*g* for 20 min. The supernatant contained the cytoplasmic fraction. Cell lysates were irradiated and checked for any live bacteria after plating. Cell lysates were used for measuring protein concentration. Samples containing 10 μg of total cytoplasmic proteins were separated by gel electrophoresis and transferred to nitrocellulose membranes. The membranes were blocked overnight in 1× Tris‐buffered saline (TBS) containing 0.1% Tween‐20 and 3% bovine serum albumin at 4°C. The membranes were washed extensively with 1× TBS buffer and then probed with anti‐LC3 or anti‐p62 polyclonal antibody followed by horseradish peroxide‐conjugated secondary antibody (goat anti‐rabbit). After additional rinsing with 1× TBS buffer, the membranes were exposed to a chemiluminescent substrate in the presence of hydrogen peroxide, using Immun‐Star WesternC Chemiluminescent kit (BioRad). A VersaDoc Model 4000 (BioRad) imaging system was used to capture the image.

### 2.5 Cell culture

#### 2.5.1 Cell lines and PBMCs

HeLa and J774A.1 cells were grown with DMEM plus 10% FBS, and spleen cells obtained from BALB/c mouse were grown with RPMI 1640 plus 5% FBS. J774A.1 or HeLa cells (3‐4 × 10^6^cells/mL) were grown in a 10 mL culture tube with appropriate culture media for 2 h. First we examined the effect of rapamycin treating the cells in the absence presence of varying concentration of Rapamycin (10‐100 μM) for 30 min followed by infection. In subsequent experiment cells were infected with either with *B. thailandensis* or *B. pseudomallei* strains BpK96243, Bp406e, or BpMSHR305 (10 moi) and *B. mallei* strain, Bm FmH (10 moi) in the presence or absence of Rapamycin (50‐100 μM) for 1‐2 h. The cells were spun down and the supernatant was discarded. The cells were refeed with fresh culture medium and grown for 24 h at 37°C, CO_2_ incubator. Culture conditions and irradiation methods for ensuring safe use in the BSL2 lab were carried out as described elsewhere.^21^, ^22^ Peripheral blood mononuclear cells (PBMCs) were obtained from consenting healthy donors in accordance with an Institutional Review Board‐approved research donor protocol. PBMCs were isolated by standard density gradient centrifugation with Ficoll‐Hypaque, harvested from the interface, washed, and re‐suspended in RPMI 1640 medium (Invitrogen, Carlsbad, CA) as described previously^23^ and used for *B. pseudomallei* infection to characterize autophagy biomarkers p62/NBR1.

#### 2.5.2 Autophagy biomarkers

J774.1, HeLa, or PBMCs were treated with or without rapamycin for activation of autophagy. After 30 min rapamycin treatment, cells were infected and harvested after 20 h. Cells were treated with 400 μL of RIPA cell lysis buffer 2 (Enzo Life Sciences Inc., Farmingdale, NY) containing PMSF (1 mM final, Thermoscientific, IL, USA), DNase (20 μg/mL final, Thermoscientific) and protease inhibitor cocktails (5 μL, Thermoscientific). Mouse spleen cell lysates were prepared as previously described in ref.^22^ Briefly, individual spleens were suspended and disaggregated in a 60 × 15 mm petri dish (Falcon 351007, Becton‐Dickinson, Franklin Lakes, NJ) in 3 mL of wash medium RPMI 1640 medium (Life Technologies, Grand Island, NY) containing 25 mM HEPES and 2 mM glutamine. After allowing the large debris to settle, an aliquot of the extract was collected and stored at −70°C until it was irradiated to sterilize the sample before transfer from BSL‐3 to BSL2. Part of the extract before irradiation was used to make 10‐fold dilutions in sterile water to determine the number of colony‐forming units on sheep blood agar plates that were incubated at 37°C for 2‐3 days. Cells with lysis buffer were incubated on ice for 60 min. The cytoplasmic fraction (aqueous phase) was collected by centrifuging the lysates at 10 000*g* for 10 min. The collected proteins were stored at −70°C until they were irradiated and ready to be used for p62 and NBR1 assays.

#### 2.5.3 Cell infections

Cells (J774.1, HeLa, spleen cells or PBMCs) (3 × 10^6^‐4 × 10^6^ cell/mL) were cultured in 2 mL MEM supplemented with 5% FBS in a 10 mL culture tube and treated with Rapamycin (50 μM) for 30 min. Cells were infected for 1 h, washed twice with culture medium and treated with Gentamicin (50 μg/mL) for 1 h. One hour post‐Gentamicin treatment cells were washed twice with culture medium and refeed with fresh medium containing rapamycin and incubated for 0 and 20 h. Infected cells were harvested at 0 and 20 h time points and treated with 300 μL of RIPA cell lysis buffer two containing PMSF, DNase, and protease inhibitor cocktails. Cell lysates (10 μL) were serially diluted (1:10) with deionized water, and 100 μL of the diluted lysate was plated onto sheep blood agar plates for colony forming units (CFU), and plates incubated at 37 C for at least 2–3 days before counting colonies. The remaining lysates was processed for protein estimation and used for p62 and NBR1 assay.

#### 2.5.4 p62 assay

Cell lysates at different protein concentration were used for p62 assay according to the manufacturer's protocol. Briefly, samples and standards were added to wells coated with a monoclonal antibody specific for p62 and then incubated. After washing the plate, rabbit polyclonal antibody to p62 was added and then incubated. After washing, the enzyme (HRP)‐conjugated anti‐rabbit IgG was added and incubated. Finally, a color developer substrate (TMB) was used to activate the enzyme reaction. After 30 min incubation of TMB, stop solution was added to each well and the plate absorbance was read in a spectrophotometer at 450 nm. The amount of p62 was calculated from the plot of standard curve of human p62 standards according to the manufacturer's protocol.

#### 2.5.5 NBR1 assay

Cell lysates at various protein concentrations were used for NBR1 assay according to the manufacturer's protocol. Briefly, samples and standards were added to wells coated with a monoclonal antibody to NBR1 and then incubated. After washing, the enzyme (HRP)‐conjugated monoclonal antibody to NBR1 was added and incubated. Finally, a color developer substrate (TMB) was used to activate the enzyme reaction. After 30 min incubation of TMB, stop solution was added to each well and the plate absorbance was read in a spectrophotometer at 450 nm. The amount of NBR1 was determined from the plot of standard curve of human NBR1 standards.

### 2.6 Immunofluorescent microscopy

Formalin‐fixed paraffin embedded (FFPE) mouse tissue sections were deparaffinized using xylene and a series of ethanol washes. After 0.1% Sudan black B (Sigma) treatment to eliminate the autofluorescence background, the sections were heated in Tris‐EDTA buffer (10 mM Tris Base, 1 mM EDTA Solution, 0.05% Tween 20, pH 9.0) for 15 min to reverse formaldehyde crosslinks. After rinses with PBS (pH 7.4), the section were blocked with PBS containing 5% normal goat serum overnight at 4°C. The sections were incubated with Rabbit anti‐NBR1 (1:100) and Rat anti‐Mouse CD45 (1:200) for 2 h, 22°C. After rinses with PBS, the sections were incubated with secondary goat anti‐rabbit Alex Fluor 488 (green, 1:500) and goat anti‐rat Alex Fluor 561 (red, 1:500) antibodies for 1 h, 22°C. For *B. mallei* detection the tissue section after blocking with goat serum were incubated with rabbit polyclonal anti‐B. mallei (GB18; 1:500; a kind gift from Dr. Jenny Chua and Dr. David Waag, USAMRIID) for 2 h at 22°C. After rinses with PBS, the sections were incubated secondary goat anti‐rabbit Alex Flour 561 (red, 1:500) antibodies for 1 h at 22°C. Sections were cover slipped using the Vectashield mounting medium with DAPI (Vector Laboratories). Images were captured on a Zeiss LSM 880 confocal system and processed using ImageJ software.

#### 2.6.1 In situ hybridization (ISH)

In situ hybridization was performed using RNAscope RED 2.5 kit (Cat# 322360) by Advanced Cell Diagnostics (ACD). Briefly, 20 ZZ probes against mouse SQSTM1/p62 (Cat# 444221) were synthesized and purchased from ACD. After deparaffinization and peroxidase blocking, sections were covered with ISH probes and incubated at 40°C in hybridization oven for 2 h. They were rinsed and the ISH signal is amplified by applying Pre‐amplifier and Amplifier conjugated with HRP. A red substrate‐chromogen solution was applied for 10 min at 22°C. The slides were further stained with hematoxylin, air dried, and mounted.

### 2.7 Statistical analysis

Data were analyzed for statistical significance of each treatment and repetition of the experiment was analyzed by the equation relating the target protein concentration to the total protein as

$T\,\arg et\;Protein=\alpha^{*}{T\,\arg et\;Protein}^{\beta }$

The ratio of target to Total protein is inferred from the equation at the point where the Target protein had reached half its maximal concentration, this being a region where the ratio was most nearly constant. That is, the log ratio is estimated as:

$L\mathrm{og}\left(Ratio\;T\,\arg et\;to\;Total\right)=\log\left({T\,\arg et}_{\%50}\right)-\left((\log(0.5)-\alpha )\text{/}\beta \right).$

A two‐way ANOVA was fit within each time point, where the factors being tested were rapamycin concentration (DMSO, 50 or 100 μM) and *B. thailandensis* or *B. pseudomallei* bacteria (present or not). The two‐way ANOVA test was used to determine the statistical significance of the effect of Rapamycin treatment on bacterial infection following standard ANOVA procedures. Confidence intervals and standard errors were taken from asymptotic normal methods. Estimates were obtained in SAS proc NLMIXED SAS version 9.4.

## 3 RESULTS

### 3.1 Autophagy reduced intracellular p62/NBR1 and survival of *thailandensis* in infected J774A.1 cells

**Figure 1:**
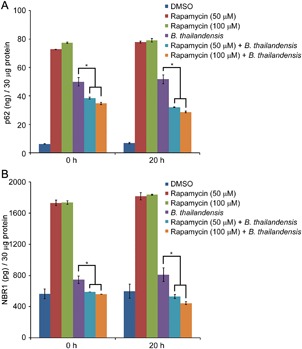
Autophagy reduced intracellular p62/NBR1 and survival of *B. thailandensis* in infected cells. J774A.1 cells (3 × 10^6^cells/mL) were first treated with Rapamycin (50 and 100 μM) for 30 min and then infected with *B. thailandensis* (20 MOI) for 2 h. After infection the cells were washed, pelleted, lysed, and labelled as 0 h. The whole cell lysates were measured for protein concentration and 30 μg protein were used to measure p62 and NBR1 as described in experimental procedures. Infected cells that were incubated for overnight, pelleted, lysed, and labeled as 20 h. Cell lysates 30 ug were used for p62 and NBR1 assay and a part of it plated at different dilutions for bacterial colony count (Table 1). Data presented from two independent experiments. Two‐way ANOVA test was performed at each time point for analyzing the effect of rapamycin and *B. thailandensis*. For p62, the *P*‐value at 0 h of rapamycin treatment and effect of concentration are <0.0001 and 0.0091 and at 20 h of rapamycin treatment and effect of concentration are <0.0001 and 0.1057. For NBR1, the *P*‐value at 0 h of rapamycin treatment and effect of concentration are <0.0029 and 0.5224 at 20 h of rapamycin treatment and effect of concentration are <0.0085 and 0.1977. Asterisk indicates significant difference between the pairwise comparisons (*P* ≤ 0.0091)

We first examined autophagy and survival of *B.thailandensis,* a closely related species to *B. pseudomallei* in infected J774A.1 cells that were treated with rapamycin, a pharmacological inducer of autophagy. Cell lysates were prepared at 2 and 20 h post infection to measure p62/NBR1, and plated in equal amount for bacterial counts. Our results indicated that rapamycin treatment (50 and 100 μM) of *B . thailandensis* infected cells reduced intracellular p62 and NBR1 compared to no rapamycin treatment (Figure 1). By 20 h post infection rapamycin treatment also reduced the number of viable bacteria recovered for example, at 10^−4^ dilution 285 CFU and 312 CFU at 50 and 100 μM rapamycin treatment respectively compared to 545 CFU with infection no rapamycin treatment Table 1. These results indicated an intracellular reduction in p62 as well as bacterial counts (Figure 1, Table 1) and suggested that the activation of autophagy with rapamycin treatment reduced intracellular growth of *B.thailandensis* in J774A.1 cells.

**Table 1:** Stimulation of autophagy suppresses the intracellular survival of *B.thailandensis* in J774A.1 cells

Plate ID				
Cell	Rapamycin	Dilution (*B.thailandensis*)	Total no. of colonies after gentamicin treatment at 0 h	Total no. of colonies after 20 h
+	–	10^−1^	>600	>600
+	–	10^−2^	>600	>600
+	–	10^−3^	39	>600
+	–	10^−4^	2	545
+	50 µM	10^−1^	>600	>600
+	50 µM	10^−2^	123	>600
+	50 µM	10^−3^	10	>600
+	50 µM	10^‐4^	1	285
+	100 µM	10^−1^	>600	>600
+	100 µM	10^−2^	48	>600
+	100 µM	10^−3^	5	>600
+	100 µM	10^−4^	1	312

J774A.1 cells (3 × 10^6^ cells) were treated with or without Rapamycin (50 and 100 μM) for 30 min, then infected with *B.thailandensis* (20 MOI) for 2 h. Cells were washed with culture medium and treated with gentamicin (50 μg/mL) for 1 h. One hour post‐gentamicin treatment, cells were washed and refed with fresh culture medium containing rapamycin and incubated for 0 and 20 h. Cells at 0 and 24 h after culture were lysed, protein concentration were determined for measuring p62 and NBR1 (Figure 1) and a part of the cell lysates were plated at different dilutions to determine bacterial colony counts (0 and 20 h). Data represents the colony forming units (CFU) at multiple dilutions with or without rapamycin treatment.

### 3.2 Autophagy reduced intracellular p62/NBR1 and survival of *pseudomallei* in infected HeLa cells

**Figure 2:**
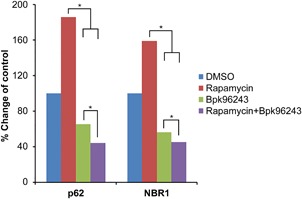
Autophagy reduced intracellular p62/NBR1 and survival of *B. pseudomallei* in infected HeLa cells. HeLa cells (4 × 10^6 ^cell/mL) were induced with Rapamycin (100 μM) and infected with BpK96243 (10 MOI) for 20 h. Cells were pelleted and lysed. The whole cell lysates (30 μg) were used to measure p62 and NBR1 at multiple dilutions as described in experimental procedures. Data presented as % change of control of p62/NBR1 from the dose response curve of the combined data (Supplemental Figure S1) from two independent experiments. For statistical significance each treatment and repetition of the experiment was analyzed by the equation as described in the method section. Confidence intervals and standard errors are taken from asymptotic normal methods. For p62, pairwise comparisons shows the following *P*‐values; BpK96243 versus DMSO (0.0002), BpK96243 versus Rapamycin (<0.0001), BpK96243 versus Rapamycin+ BpK96243 (0.0004), DMSO versus Rapamycin (0.5809), DMSO versus Rapamycin+ BpK96243 (<0.0001), Rapamycin versus Rapamycin+ BpK96243 (<0.0001). For NBR1, pairwise comparisons shows the following *P*‐values; BpK96243 versus DMSO (<0.0001), BpK96243 versus Rapamycin (<0.0001), BpK96243 versus Rapamycin+ BpK96243 (0.0017), DMSO versus Rapamycin (<0.0001), DMSO versus Rapamycin+ BpK96243 (<0.0001), Rapamycin versus Rapamycin+ BpK96243 (<0.0001). Asterisk indicates significant difference between the pairwise comparisons (*P* ≤ 0.0017)

Next we examined *B. pseudomallei* infection of HeLa cells stimulated with rapamycin. We noted a dose‐dependent decrease in p62 with *B. pseudomallei* infection and rapamycin treatment compared to *B. pseudomallei* infection only with no rapamycin treatment (Supplemental Figure S1). Similar results were observed with NBR1 but at a lesser level (Supplemental Figure S1). Overall our results showed a siginificant decrease in intracellular p62 and NBR1 with rapamycin treatment followed by *B. pseudomallei* infection compare to *B. pseudomallei* infection only (Figure 2).

### 3.3 Autophagy and bacterial persistence in a mouse glanders model

**Figure 3:**
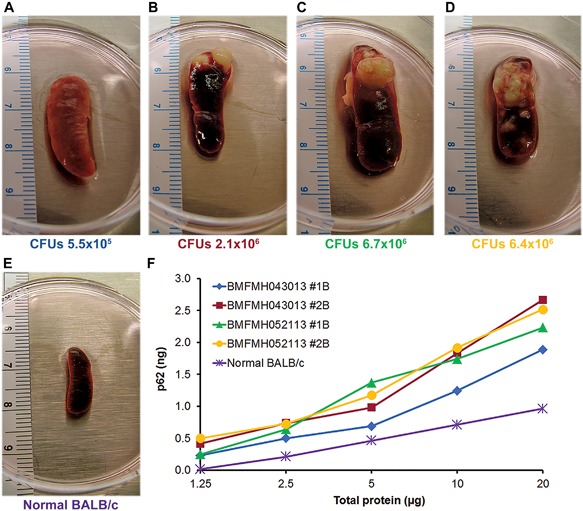
Autophagy and bacterial persistence in a mouse glanders model. Aerosol exposure of BALB/c mice at varying concentrations of *B. mallei* FMH were performed as described in experimental procedures. Bacterial exposure up to 21 days considered as acute phase and post 21‐60 days as considered as chronic phase. Spleens were isolated and measured size of the spleens at 27d (blue [A] and dark red [B]) and at 47d (green [C] and yellow [D]). Spleen from individual mouse was homogenized for purifying spleen cells and lysed for preparing lysates to determine bacterial count after plating and to measure p62 levels as described in section 2. (A–D) Spleens from aerosol exposed mice with bacterial count indicated below, and (E) spleen from normal BALB/c mice. (F) p62 levels in spleen cell lysates of *B. mallei* exposed to individual mouse at two different time points as indicated above

While previouss investigations were undertaken to assess the suitability of BALB/c and C57BL/6 mice with *B. pseudomallei* infection as animal models for the different forms of human melioidosis, there is very limited data from cell culture models of infection with *B. mallei*.^24^ Furthermore, in the context of the potential use as a biothreat agent with aerosol exposure, the role of autophagy in clearing *B. mallei* infection has not been studied. To examine the role of *B. mallei* infection in stimulating autophagy and pathogen clearance from the host, we infected BALB/c mice with *B. mallei* strain FMH23344 by aerosol exposure and examined spleens at different time points for viable bacteria and intracellular p62 levels. A massive swollen spleen with a large granulomatous growth was observed with *B. mallei* infection 27d (Figures 3A and 3B) and 47d post infection (Figures 3C and 3D) in comparison to uninfected spleen (E). Intracellular persistence of *B. mallei* was observed in infected spleens 27d post infection (Figures 3A and 3B) and bacterial counts increased 47d post infection (Figures 3C and 3D). In spleen cell lysates of the individual mouse in comparison to uninfected spleen cells (E), a dose dependent increase in p62 levels was also observed both 27d and 47d post infection (Figure 3F). These results indicated a 4‐5 fold increase in p62 in spleen cells with *B. mallei* infection as compared to uninfected mice, correlating elevated p62 with increased bacteria.

### 3.4 Autophagy and bacterial persistence in a mouse melioidosis model

**Figure 4:**
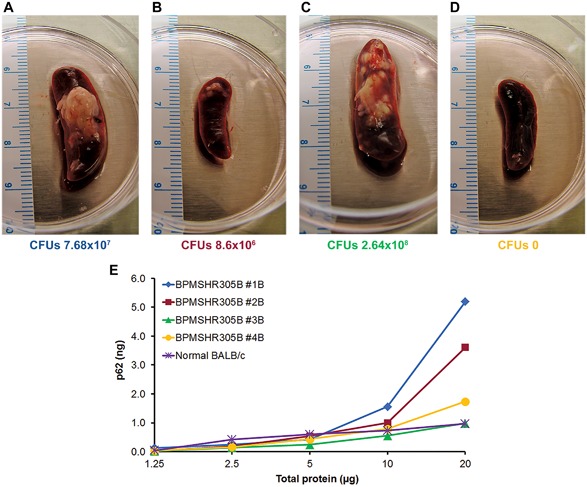
Autophagy and bacterial persistence in a mouse melioidosis model. Aerosol exposure of BALB/c mice at varying concentrations of *B. pseudomallei* MSHR305 were performed as described in section 2. Spleens were isolated at day 35 and measured size of the spleens. Spleens from individual mice were homogenized to prepare spleen extracts and cfu determined. p62 levels were measured as described in section 2. (A–D). Spleens of *B. pseudomallei* with pyogranulomas and persistence of bacteria in chronically infected mice are indicated below; (E) p62 levels in spleen cell extracts from individual mice as shown in A–D (same color)

**Figure 5:**
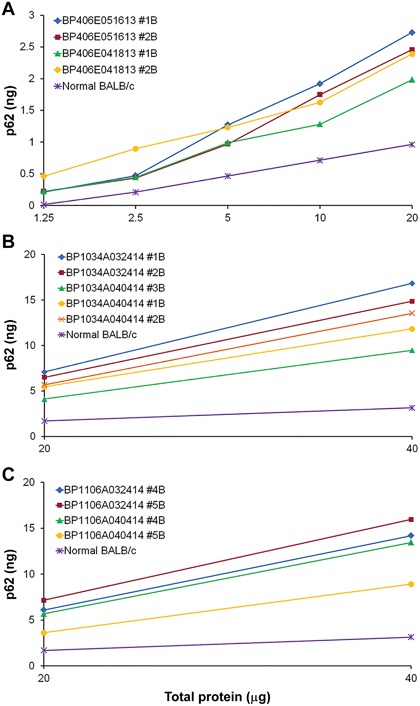
*B. pseudomallei* strains Bp406e, Bp1106A, Bp1034a exposure increased p62 in spleens of BALB/c mice. *B. pseudomallei* strain (a) BP406e; (b) Bp1034a and (c) BP1106a were used to expose/infect BALB/c mice as indicated below. Spleens from individual mice were homogenized to prepare lysates for measureing p62 levels or plated for colony count as described earlier. Data presented as p62 levels in spleen cell lysates from individual mice at different time points. (A) BP406e aerosol infection and p62 (pg) at different concentrations 1.25 to 20 μg of total protein at days (34 to 62) post infection (mice #1B at 34d and at 62d; mice #2B at 34d and 62d); (B) Bp 1034a ip infection and p62 (pg) at 20 and 40 μg of total protein at 3 or 14 days post infection (mice #1B, #2B,#3B at 3d and #1B, #2B at 14d); (C) BP1106a ip infection and p62 (pg) at 20 and 40 μg of total protein at 3 or 14 days post infection (mice #4B, at 3d and 14 and mice #5B, at 3d and14d)

*B. pseudomallei* growing in cell culture infections are susceptible to killing by autophagy and display reduced survival if autophagy is pharmacologically induced above the basal level by rapamycin treatment.^15^ Our earlier data also showed that autophagy induction with *B. pseudomallei* infected HeLa cells reduced bacterial counts (Figure 1, Table 1). However, it remained unclear whether *B. pseudomallei* infection of mice could induce autophagy and if this was sufficient to clear the infection. To examine this, we infected BALB/c mice with virulent *B. pseudomallei* MSHR305 and analyzed intracellular p62 and bacterial loads in spleens. Similar to *B. mallei* infections, a massive swollen spleen with a large granulomatous growth was observed with *B. pseudomallei* at 35d post infection (Figures 4A‐D). In comparison to uninfected spleen, a dose dependent increase in p62 was observed in cell lysates of all individual mice at 35d post infection (Figure 4E), except for mouse #3B and #4B. While mouse #3B had a significant bacterial load, both mouse #3B and mouse#4B had p62 levels that were barely detectable and similar to uninfected control. These in vivo results with *B. pseudomallei* were not as consistent as those obtained with *B. mallei* in BALB/c mice (Figure 3). For further confirmation, we examined p62 and bacterial counts in spleen cell lysates of BALB/c mice after aerosol exposure or intraperitoneal (i.p.) infections. BALB/c mice were infected by aerosol exposure with Bp 404e or i.p. infected with Bp HBPUB10134a, and Bp 1106A. Spleens were collected from these mice at 34d and 62d post exposure to Bp 406e, and 3d and 14d post infection to Bp 1106A, and Bp HBPUB10134a, respectively. Spleen lysates of most mice showed an overall 2‐5 folds increase in p62 levels, and variable bacterial counts (cfu) regardless of the route of infection (Figure 5A–C, and Table 2), suggesting that similar to *B. mallei* infections, functional autophagy was reduced by *B. pseudomallei* infection of BALB/c mice leading to an accumulation of p62. We also examined intracellular p62 in spleen cells of C57BL/6 mice after 30 and 56 days post infection, which showed an increase in p62 protein in infected mice as compared to non‐infected mice and an increase of p62 with time (data not shown).

**Table 2:** Bacterial colony count at different time points in spleens of BALB/c mice exposed (aerosol/ip infection) to different strains of *B. pseudomallei*

*B. pseudomallei* (Aerosol/ip infection)	Spleen collected at days post infection	Mouse #	Bacterial colony count (CFU)
Bp 406e (aerosol exposure)	34	1B	1.08 × 10^9^
	62	2B	8.0
	34	1B	0
	62	2B	0
Bp1034A (ip infection)	3	1B	6.0 × 10^5^
	3	2B	1.75 × 10^5^
	3	3B	0
	14	1B	0
	14	2B	1.56 × 10^8^
Bp1106A (ip infection)	3	4B	10
	3	5B	50
	14	4B	30
	14	5B	165

*B. pseudomallei* strain BP406E, Bp1034a, and BP1106a were used to expose/infect BALB/c mice as described in Figure 5. Spleens from individual mice at different time points were homogenized to prepare lysates for measuring p62 levels as shown in Figure 5 and a part of the spleen lysates were used for plating to determine bacterial colony count. Data represent colony forming units (CFU) in spleen cell lysates from individual mice at different time points.

### 3.5 p62 and LC3‐I increased in spleen cells of mice infected with *mallei* or *B. pseudomallei*

**Figure 6:**
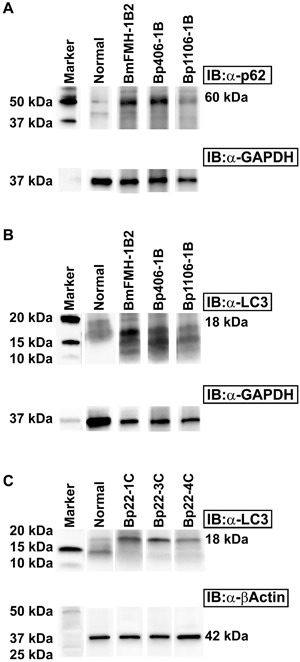
p62 and LC3‐I increased in spleen cells of mice infected with *B. mallei* or *B. pseudomallei*. Uninfected, *B. mallei* FMH, *B. pseudomallei* strain Bp406, Bp1106, and Bp22 (formerly, Bp KHW) infected spleen cell lysates of BALB/c and C57BL/6 mice were used for detecting p62 and LC3. Samples containing 10 μg of total proteins were separated by SDS‐PAGE gel electrophoresis and transferred to nitrocellulose membranes and then probed with anti‐LC3 or anti‐p62 polyclonal antibody followed by horseradish peroxide‐conjugated secondary antibody (goat anti‐rabbit). A, p62 50 kDa band detected after probing with anti‐p62 antibody in BALB/c mice. B, Immunoblot with anti‐LC3 antibody, the major 18 kDa LC3‐I and less 16 kDa kDa LC3‐II form protein was detected in BALB/c mice. C, Expression of LC3 in *B. pseudomallei* 22 infected C57BL/6 mice

**Figure 7:**
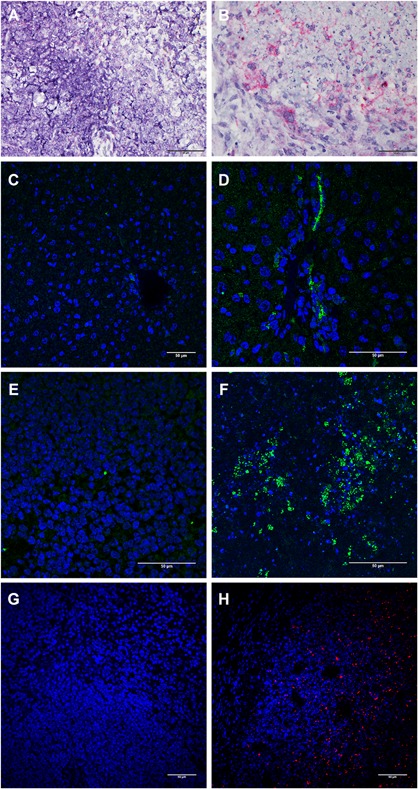
p62 upregulated in spleen and liver of *B. mallei* infected mouse. In situ hybridization was performed using synthesized 20 zz probes of mouse SQSTM1 /p62 (Cat# 444221) as described in section 2. Briefly, after deparaffinization and peroxidase blocking, sections were covered with ISH probes and hybridized as described in section 2. (A,B) In comparison with the low level of p62 mRNA (red, A) detected in the uninfected control spleen, high levels of SQSTM1/p62 (red, B) mRNA was detected in the edge region of a pyogranuloma of *B. mallei* infected spleens. (C,D) Low level of NBR1 (green) was detected in uninfected control liver (C), whereas high levels of NBR1 (green) was detected in the focus of histiocytic infiltrate of liver from a *B. mallei* infected mouse (D). (E,F) NBR1 was undetectable in the uninfected control spleen (E), but high levels of NBR1 (green) was detected in the spleen of a *B. mallei* infected mouse (F). (G,H), *B. mallei* was detected in the tissue sections of spleen after staining with Rabbit anti‐B mallei polyclonal antibody followed by incubation with secondary goat anti‐rabbit Alex Fluor 561 antibodies. G, *B. mallei* was not detected in an uninfected spleen, (H) high levels of *B. mallei* (red) was detected in the spleen of a *B. mallei* infected mouse

Ubiquitylated proteins are bound by p62 and LC3 to target protein aggregates for lysosomal degradation during autophagy, and p62 is also reduced to low levels during this process. The characteristic conversion of the cytosolic form of LC3 (LC3‐I, 18 kDa) to an autophagosomal‐associated form (LC3‐II, 16 kDa) can be used to monitor autophagy, and is a specific marker for the formation of autophagosomes. Therefore, we examined the conversion of LC3‐I to LC3‐II to confirm that the increased accumulation of cytosolic p62 in *B. mallei* or *B. pseudomallei* infected mouse spleen was due to an autophagy deficiency. The p62 (60 kDa) protein was elevated (Figure 6A, and supplemental Figure S2, panel A) in Western blots of spleen cell extracts from BALB/c mice exposed to *B. mallei* or *B. pseudomallei*, and these cells predominantly expressed LC3‐I (18 kDa) as compared to LC3‐II (16 kDa) (Figure 6B, and supplemental Figure S2, panel B). Similarly C57BL/6 mice infected with the more recently isolated *B. pseudomallei* 22 Singapore strain also showed mostly LC3‐I and barely detectable levels of LC3‐II protein (Figure 6C, and supplemental Figure S2, panel C). These results were consistent with the p62 assay data shown in Figures 4 and 5 and demonstrate that the increased cytosolic form of LC3‐I was not associated with autophagolysomal degradation of *Burkholderia*. While expression of p62/NBR1 was undetectable in uninfected mouse spleen, high levels of NBR1 were apparent in pyogranulomas of spleen and liver tissues from the *B. mallei* infected mice (Figures 7B and 7F). Additionally, high levels of NBR1 were also detected in the foci of histiocytic infiltrates in livers of *B. mallei* infected mice (D). Consistently, *B. mallei* was also detected in the spleen tissues of infected mice (H). These results suggest that an increase in p62/NBR1 levels and dissemination of *B. mallei* in different organs of infected mice linked to deficiency in functional activation of autophagy.

### 3.6 Stimulation of autophagy reduced intracellular p62/NBR1 in *pseudomallei* infected human PBMC

**Figure 8:**
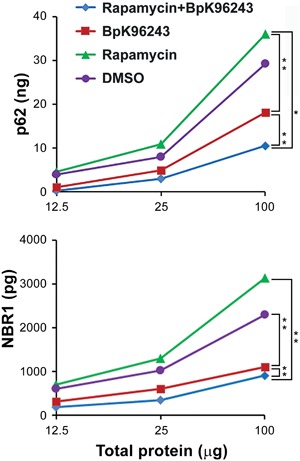
Stimulation of autophagy reduced intracellular p62/NBR1 in *B. pseudomallei* infected human PBMC. PBMCs (3 × 10^5^ cells) were were first treated with Rapamycin (100 μM) for 30 min and then infected with *B. pseudomallei* BpK96243 (10 MOI) for 20 h. Cells were pelleted and lysed. The whole cell lysates at different concentrations (12.5, 25, and 100 μg of total protein) were used for measuring p62 and NBR1 as described in section 2. Data presented as average from two independent experiments p62/NBR1 present in the total protein as indicated (12.5, 25, and 100 μg of total protein). For statistical significance each treatment and repetition of the experiment was analyzed by the equation relating the target protein concentration to the total protein as Target Protein = α *Total Proteinβ as described in section 2. Two‐way ANOVA test was performed at each time point for analyzing the effect of rapamycin and *B. pseudomallei* BpK96243. For p62, and NBR1 the *P*‐value with pairwise comparisons and asterisk indicates significant difference between these pairwise comparisons (*P* ≤ 0.0084)

We extended our study to human primary cells to compare results to the mouse and cell line infection models. Similar to the ex vivo results, PBMC treated with rapamycin or infected with Bpk96243 exhibited elevated p62 (32 and 18 ng, respectively), while rapamycin treatment of Bpk96243 infected cells decreased p62 (10 ng; Figure 8, upper panel). Similar results were observed with NBR1 (Figure 8, lower panel). These results in human primary cells demonstrate that *B. pseudomallei* infection with rapamycin treatment decreased intracellular p62 as well as NBR1 compared to controls.

## 4 DISCUSSION

Cellular autophagy is one arm of innate immune defenses that is used against invasive intracellular pathogens. In this study, we have characterized the consequence of a host key cargo adaptor protein, p62/NBR1, associated with autophagic flux with *Burkholderia* infection which initially identified as a bridge between LC3 and polyubiquitylated protein aggregates destined for autophagic removal. Our in vitro data in a cell‐culture based infection assays demonstrated that induction of autophagy with rapamycin treatment reduced intracellular p62 /NBR1 and bacterial survival in J774.1 cells infected with *B. thailandensis,* a correlation of p62/NBR1 with bacterial survival. Rapamycin treatment of HeLa cells and *B. pseudomallei* infection of HeLa cells also showed reduced levels of p62/NBR1. However in vivo *B. mallei* and *B. pseudomallei* infection of mice showed 2‐5 folds increase in p62 level with increased persistence of bacteria in spleens, which suggests in vivo functional deficiency of autophagy with pathogenic *Burkholderi* infection. In this study, our results demonstrated that in the event of autophagy deficiency with *B. mallei* or *B. pseudomallei* infection, p62 accumulates in the cytoplasm that correlates to the persistence of bacteria.

In most cases, autophagic responses leading up to xenophagy which is the engulfment of cytosolic microbes into double membrane autophagosome or LC3‐associated phagocytosis (LAP) are guided by several pathogen recognition receptors (PRRs), such as sequestosome 1/p62‐like receptors (SLRs). The SLR like p62 has a dual functionality as both a scaffold protein, which contains one or more cargo recognition domains that bind ubiquitin, and aids in trafficking for protein degradation. Thus, p62 provides a key link between the ubiquitin‐proteosome system (UPS) and autophagy by facilitating autophagic degradation of ubiquinated proteins, and decreasing aggregation of misfolded and non‐functional proteins within the cells, including bacterial pathogens, resulting in enhanced cellular survival characteristics. In the autophagolysome‐mediated degradative pathway the multi‐domain containing cargo receptor p62 functions as a signaling hub and plays a critical role in autophagy flux in clearing intracellular pathogens. Thus, the p62 protein level may be an indicator of autophagy flux with *B. mallei and B. pseudomallei* infection because p62 accumulates when autophagy is inhibited, and decreased levels can be observed when autophagy is induced.^25^, ^26^ Antigen presentation, innate immune signaling, and pathogen degradation may all involve autophagosome recruitment and activity, which play an important role in the induction of total immunity and defense against infectious diseases including *Burkholderia* infection. *B. pseudomallei* infection is known to be capable of evading autophagic killing, however, our in vitro results showed the induction of autophagy with rapamycin treatment resulted a decrease in intracellular bacterial survival and the levels of p62. But when mice were exposed to *B. mallei* or *B. pseudomallei* a noticeable lack of autophagy mediated clearance of bacteria with concomitant increase in p62 was observed.

Clinical disease of melioidosis presents a broad spectrum of severity ranging from acute fulminating sepsis, which carries a high mortality rate, to chronic persistent infection. While the acute and chronic forms of human melioidosis have been successfully modeled in mice,^24^ however, little is known about what comprises bacterial persistence in the host. A variety of host machanisms exist for recognizing and targeting intracellular bacteria to autophagosomes.^27^, ^28^ Several intracellular bacteria have evolved ways to manipulate, inhibit, or avoid autophagy in order to survive within the cell (27). BopA an effector protein secreted *B. pseudomallei* via type three secretion system has been shown to play a pivotal role in their escape from autophagy.^28^ A recent study also demonatrated that induction of autophagy in U937 infected with *B. pseudomallei* is associated with the increase of colocalization between bacteria and LC3 and LC3‐bacterial protein interaction was detected by LC‐MS/ MS analysis.^29^ In the present study our results indicate that in acute and chronic *B. mallei* or *B. pseudomallei* infected mice p62 levels were elevated, and the lack of characteristic conversion of LC3 protein from LC3‐I (18kDA) to reduced levels of LC3‐II (16 kDa) present in spleens of infected mice was indicative of autophagy deficiency. Thus, this also demonstrates, that both in susceptible and partially resistant C57BL/6 mice, the accumulation of p62 suggest that bacterial persistence was linked to lack of autophagy activity. Host innate immune system that utilizing autophagy process use double‐membrane vesicles to deliver cytoplasmic contents to lysosomes for degradation to protect against infectious pathogens. Thus, activation of autophagy process enhances the clearance of toxic, cytoplasmic, aggregate‐prone proteins, and infectious agents in which p62 itself is degraded, and remains at low levels untill autophagy is induced. It accumulates when autophagy is deficient. Previous studies demonstrate that rapamycin treatment inceased p62 levels in different cell lines and may have enhanced effects on autophagy.^30^, ^31^, ^32^, ^33^ In agreement with these previous reports our results demonstrate an increase in p62 with rapamycin treatment and this increase in p62 levels dropped significantly with *Burkholderia* infection in vitro compared to only *Burkholderia* infection. In our ex vivo infection assay it was also observed that activation of autophagy by rapamycin treatment decreased p62/NBR1 in *B. pseudomallei* infected human PBMCs. However, in vivo infection of *B. mallei* or *B. pseudomallei* p62 remained high concomitant with bacterial persistence in spleens and liver tissues of infected mice, suggesting lack of fully functional activation autophagy in clearing the pathogen.

In melioidosis patients, relapse of disease is not uncommon^34^, ^35^ illustrating poor prognosis for effective therapy. Dysfunction of p62 is also involved in the pathogenesis of human diseases, for example, aberrant accumulation of p62‐ positive aggregates structures has been detected in patients with liver disorders, tumors, and neurodegenerative diseases.^36^ It has also been shown that *B. cenocepacia* (*B. cepacia*) infected cystic fibrosis patients that leads to severe lung inflammation and lung tissue destruction (37). Defective autophagy allows *B. cepacia* to survive and replicate in ΔF508 macrophages, of which the CFR F508 mutation is the most common in cystic fibrosis patients.^37^ In macrophages where p62 is elevated, other cell components clump together, causing disruption to the autophagy process. In our ex vivo infection assay it was observed that activation of autophagy by rapamycin treatment decreased p62/NBR1 in *B. pseudomallei* infected human PBMC. *B. pseudomallei* is responsible for a number of significant human cases and apparently the lack of effective diagnosis of melioidosis delays proper treatment. In the mouse model of *Burkholderia* infection with various strains of *B. pseudomallei and B. mallei*, our study suggests that an increase in cytosolic p62 levels over the normal non‐infected control were consistent with bacterial burden, thus, it raised the possibility that p62 may be a potential prognostic host cell biomarker for *Burkholderia* infection. Furthermore, identifying autophagy manipulation may potentially aid to therapeutic approach for complete clearance of the pathogen. Our ongoing effort is to extend this study to examine p62 in PBMCs of human melioidosis patients is underway.

## CONFLICT OF INTEREST

The authors declare no competing financial interests.

## AUTHORS’ CONTRIBUTION

KA and JD involved in preparing bacterial cultures, infection of mouse with aerosol or ip exposure to bacteria; XZ performed the immunohistochemistry experiment; RGU contributed to study design, and analyzed data; KUS and KA designed and supervised the whole study, compiled data, drafted the manuscript text with all the authors contributing to the writing process and in reviewing the final manuscript.

## HUE

Research on human subjects was conducted in compliance with DoD, Federal, and State statutes and regulations relating to the protection of human subjects, and adheres to principles identified in the Belmont Report (1979). All data and human subjects (human PBMCs utilized for this study was isolated from blood of research were gathered and conducted for this publication under an IRB approved Phlebotomy protocol, number FY05‐05).

## Supporting information

Additional Supporting Information may be found online in the supporting information tab for this article.

**Figure S1**. Stimulation of autophagy with rapamycin treatment followed by B. pseudomallei infection decreased intracellular p62 as well as NBR1
**Figure S2**. p62 and LC3 increased in spleen cells of mice infected with B. mallei or B. pseudomallei
