# Supplementary material for: An increase in intracellular p62/NBR1 and persistence of Burkholderia mallei and B. pseudomallei in infected mice linked to autophagy deficiency
Source: Immun Inflamm Dis. 2018 Dec 19;7(1):7–21. doi: 10.1002/iid3.239 (PMC6416765; doi:10.1002/iid3.239)
Supplement: Supplementary file 1 — Figure S1. Stimulation of autophagy with rapamycin treatment followed by B. pseudomallei infection decreased intracellular p62 as well as NBR1 Figure S2. p62 and LC3 increased in spleen cells of mice infected with B. mallei or B. pseudomallei [file IID3-7-7-s001.docx]

**Supplemental Figures**

**An increase in intracellular p62/NBR1 and persistence of *Burkholderia mallei* and *B. pseudomallei* in infected mice linked to autophagy deficiency**

Kamal U. Saikh^1†^, Jennifer L. Dankmeyer^2^, Xiankun Zeng^3^, Robert G. Ulrich ^1^, and Kei Amemiya^2^

^1^ Department of Immunology, and ^2^ Department of Bacteriology, ^3^ Department of Pathology, Army Medical Research Institute of Infectious Diseases, 1425 Porter Street, Frederick, MD 21702


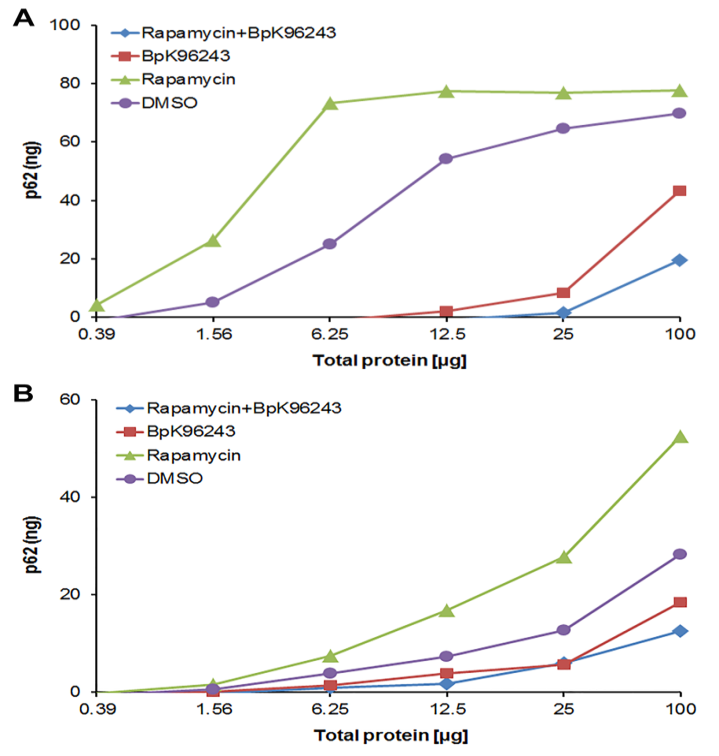

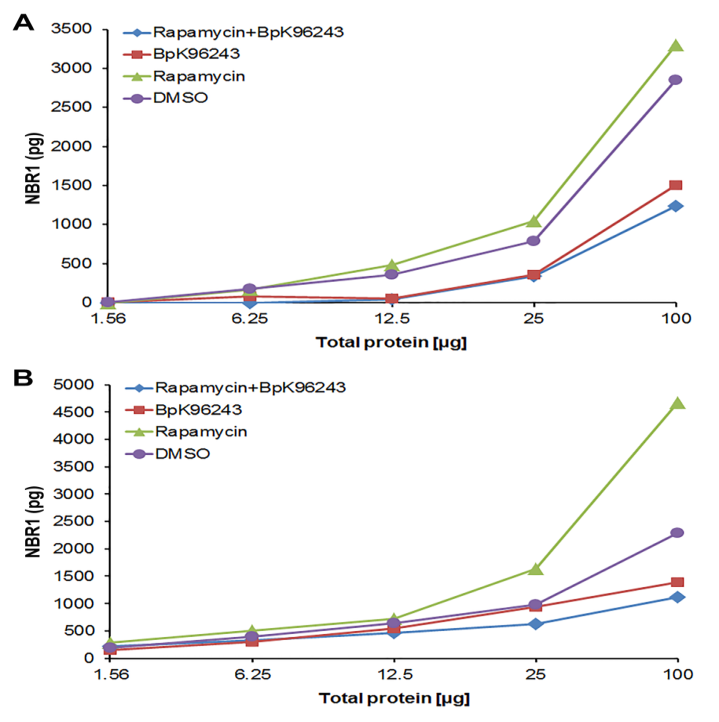


**Suppl. Fig. 1. Stimulation of autophagy with rapamycin treatment followed by *B. pseudomallei* infection decreased intracellular p62 as well as NBR1**

HeLa cells (4×10^6^cell/ml) were induced with Rapamycin (100 µM) and infected with BpK96243 (10 MOI) for 20 h. Cells were pelleted and lysed. The whole cell lysates (total protein) at different concentration were used to measure p62 and NBR1 as described in experimental procedures. Data presented as dose response curve of p62 (A and B) and NBR1 (A and B) at different concentration of total protein from two independent experiments.


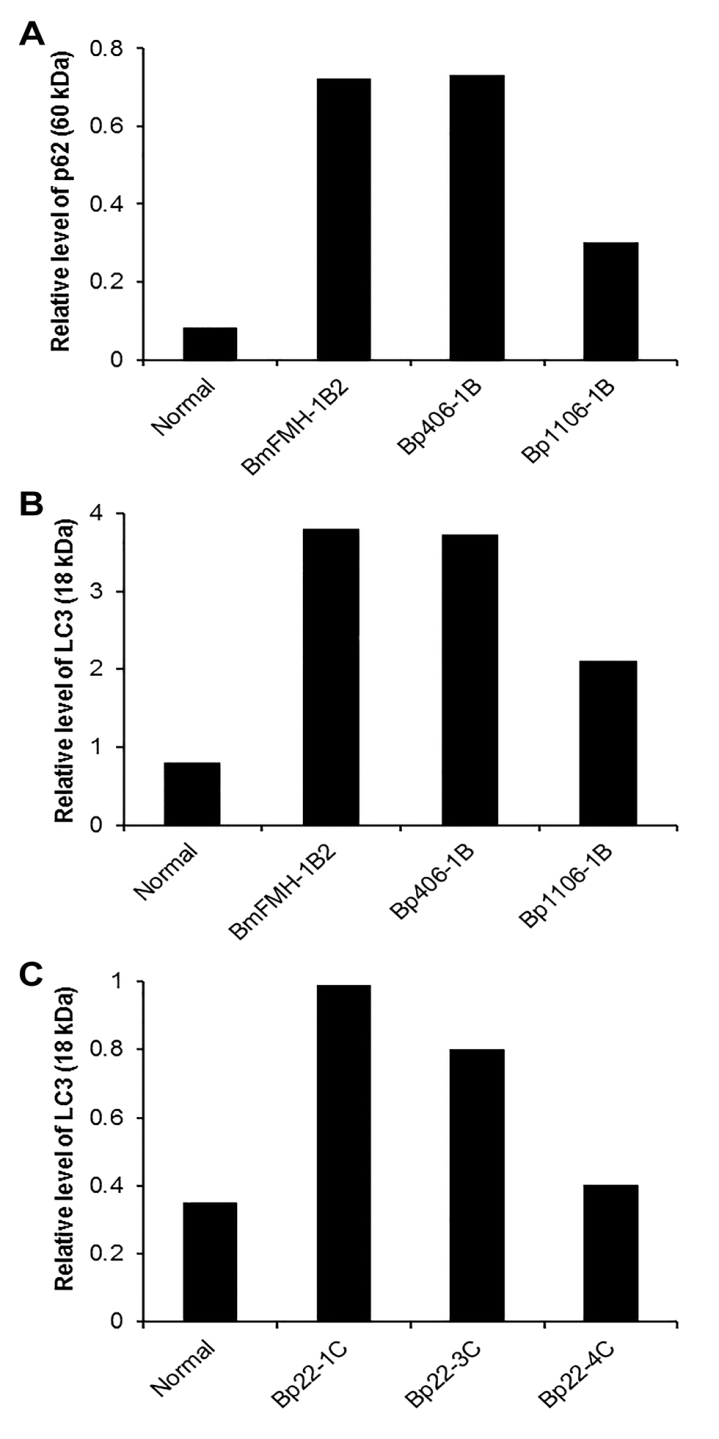


**Suppl. Fig. 2.** **p62 and LC3 increased in spleen cells of mice infected with *B. mallei* or *B. pseudomallei.***

Uninfected, *B. mallei* FMH, or *B. pseudomallei* strain Bp406, Bp1106 and Bp22 (formerly Bp KaW) infected spleen cell lysates of BALB/c (A and B), and C57BL/6 mice (C) were used for detecting p62 and LC3. Data presented as the relative levels (densitometry value) of P62 (A) and LC3-I (B and C) as shown in western blot Fig.6.
